# *Candida auris*: A Review of Recommendations for Detection and Control in Healthcare Settings

**DOI:** 10.3390/jof5040111

**Published:** 2019-11-28

**Authors:** Diego H. Caceres, Kaitlin Forsberg, Rory M. Welsh, David Joseph Sexton, Shawn R. Lockhart, Brendan R. Jackson, Tom Chiller

**Affiliations:** 1 Mycotic Diseases Branch, Centers for Disease Control and Prevention, Atlanta, GA 30333, USA; mpo6@cdc.gov (R.M.W.); ogi3@cdc.gov (D.J.S.); gyi2@cdc.gov (S.R.L.); iyn0@cdc.gov (B.R.J.); tnc3@cdc.gov (T.C.); 2 IHRC, Inc., Atlanta, GA 30346, USA

**Keywords:** *Candida auris*, diagnosis, detection, healthcare settings, infection control practices, outbreak

## Abstract

*Candida auris* is an emerging multidrug-resistant fungal pathogen. Since first reported in 2009, *C. auris* has caused healthcare outbreaks around the world, often involving high mortality. Identification of *C. auris* has been a major challenge as many common conventional laboratory methods cannot accurately detect it. Early detection and implementation of infection control practices can prevent its spread. The aim of this review is to describe recommendations for the detection and control of *C. auris* in healthcare settings.

## 1. Introduction

*Candida auris* is an emerging multidrug-resistant fungus that has caused outbreaks of invasive infections in healthcare facilities around the world. *C. auris* has been reported from dozens of countries from six continents and has caused outbreaks in places such as Colombia, India, South Africa, Spain, and the United States (https://www.cdc.gov/fungal/candida-auris/tracking-c-auris.html). Healthcare facilities have reported *C. auris* outbreaks in critically ill hospitalized patients with high crude mortality rates (30% to 72%) [1,2,3]. Risk factors for *C. auris* bloodstream infections (BSIs) are similar to the risk factors for other *Candida* species BSIs, including recent major surgical procedures, diabetes, use of broad-spectrum antibiotics, long-term hospitalizations, and the presence of devices, including breathing tubes, feeding tubes, and central venous catheters. Risk factors for candidemia differ by the population affected. For example, in the United States, patients with neurologic diseases in long-term care with many devices may be at higher risk of developing invasive *C. auris* infections [3,4]. Infections can occur in patients of all ages, but most infections have been reported in adults [4]. The ability to accurately identify *C. auris* and the capacity to implement infection control practices, including environmental cleaning, are critical to control and prevention of *C. auris* outbreaks. Here we review recommendations for detection and control of *C. auris* based on published literature and experiences of staff of the U.S. Centers for Disease Control and Prevention (CDC). 

## 2. *Candida auris* Identification 

Identification of *C. auris* isolates can be challenging, as conventional phenotypic methods for yeast identification may misidentify *C. auris* isolates as *Candida haemulonii*, *Candida sake*, *Rhodotorula glutinis*, or other *Candida* species, in part because *C. auris* is not in the databases or result options for some methods. Based on the CDC’s recommendations for the identification of *C. auris*, Table 1 describes the most common misidentifications based on frequently used yeast identification methods [5,6]. However, efforts to improve *C. auris* identification methods have made substantial progress in the last few years. The development of a high-salt, high-temperature enrichment culture-based method has made it possible to reliably isolate *C. auris* from complex sample types [7,8,9]. Once an isolate is obtained, identification of *C. auris* can be efficiently accomplished with matrix-assisted laser desorption/ionization time-of-flight (MALDI-TOF) mass spectrometry. For MALDI-TOF identification, it is important to ensure *C. auris* is in the reference database [5,6,10]. The Bruker Biotyper (Bruker Daltonik GmbH, Bremen, Germany) and the VITEK MS (bioMérieux, Marcy, L’Etoile, France) include *C. auris* in their Research Use Only and certain versions of their FDA-approved system databases [3,5,11]. If MALDI-TOF is not available, laboratories can reliably identify an isolate by sequencing the D1–D2 region of the 28s rDNA or the internal transcribed spacer (ITS) regions of rDNA [12,13,14]. 

For phenotypic yeast identification, the VITEK 2 system (bioMérieux, Marcy, L’Etoile, France) included *C. auris* in its recent software upgrade (version 8.01). Recently, a study showed this software update had limited ability to correctly identify *C. auris* from the African and East Asian clades but was able to accurately identify isolates from the South American clade [15]. Clinical validation of this VITEK 2 system upgrade is pending. Hence, all isolates identified using this system, as *C. auris*, *C. famata*, and species in the *C. haemulonii* complex should be confirmed by MALDI-TOF or DNA sequencing [3,5]. Validation of the VITEK 2 system would make *C. auris* isolate identification more accessible, as it is widely used in laboratories [6,16,17,18]. 

Beyond improved methods to identify a cultured isolate, a number of culture-independent methods for the detection of *C. auris* have been recently described. Culture-independent methods are highly attractive because results can be obtained in hours rather than days, allowing for more rapid identification of colonized patients. At the time of writing, clinical evaluations of three culture-independent tests using culture-based gold standards have been published. These include a Taqman quantitative PCR (qPCR), a SYBR green qPCR, and a T2 Magnetic Resonance assay; all performed well with clinical sensitivities and specificities close to or exceeding 90% [19,20,21,22,23]. In the United States, the Taqman-based qPCR is currently the mostly widely used culture-independent test and is employed for *C. auris* surveillance at CDC and the Wadsworth Center in New York, as well as an increasing number of Antibiotic Resistance Laboratory Network (AR Lab Network) laboratories. Recent publications have described successes adapting the Taqman qPCR to the BD Max system (Becton Dickinson, NJ, USA), which automates the test and substantially reduces associated labor [19,24,25]. Additional promising culture-independent tests have been developed, although their performance with clinical samples is not yet known [26,27,28,29,30,31]. 

All confirmed identifications of *C. auris* should be reported to local or national public health authorities, and infection control practices to prevent transmission, should be implemented at facilities where the patients reside [32,33,34].

## 3. *Candida auris* Antifungal Susceptibility Testing

Antifungal resistance of *C. auris* isolates varies across the phylogenetic clades, but multidrug-resistance is common, especially among isolates of the South Asia clade [13]. Susceptibility testing using broth microdilution for azoles and echinocandins or gradient diffusion for azoles, echinocandins, and amphotericin B are preferred. Erroneous susceptibility results have been reported for *C. auris* using the VITEK 2 for amphotericin B [6,35,36]. There are currently no established minimum inhibitory concentration (MIC) breakpoints for susceptibility of *C. auris* isolates [37]. CDC suggests the following tentative breakpoints based on the breakpoints of closely related *Candida* species and on expert opinion: fluconazole ≥32 µg/mL, amphotericin B ≥2 µg/mL (round to 2 if an MIC of 1.5 is found using Etest), caspofungin ≥2 µg/mL, micafungin ≥4 µg/mL, and anidulafungin ≥4 µg/mL [5,38].

## 4. Infection Control Practices

Implementation of infection control practices is crucial for controlling *C. auris* outbreaks in healthcare settings [2]. Lapses in infection control, delays in recognition of cases or delays in the implementation of infection control activities may result in rapid transmission of *C. auris* among patients. Some *Candida* are considered commensal organisms common in human flora, and the source of infection is generally autoinoculation, as opposed to patient-to-patient transmission. However, *C. auris* is highly transmissible among patients, perhaps due to its proclivity for persistent skin colonization [39]. Preliminary data suggest that patients who underwent placement of invasive medical devices or procedures, such as central venous catheters, were at greater risk of *C. auris* bloodstream infection compared with patients with bloodstream infections caused by other *Candida* species [1]. Because *C. auris* commonly colonizes skin, catheters may provide a means for this fungus to enter the bloodstream [40]. The transmissibility of *C. auris* is likely also driven by its ability to contaminate the patient care environment. *C. auris* has been found on healthcare surfaces and medical equipment and can persist on such surfaces for long periods [7,41,42]. Preventing spread of *C. auris* is dependent on two elements: 1) identification of cases; and 2) implementation of infection control precautions for all identified cases to minimize likelihood of transmission to other patients. For infection control purposes, a case is considered an occurrence of either *C. auris* colonization or infection in a patient [40,43]. The following recommendations for infection control are summarized in Table 2.

Identification of cases: *C. auris* is transmissible whether a patient has *C. auris* infection or colonization. Thus, infection control precautions are the same for patients with *C. auris* infection or colonization. Implementation of these practices starts with the identification of cases. The most basic type of case recognition is the identification of incident clinical cases, meaning *C. auris* through processing of routine clinical specimens. *Candida* isolates obtained from a sterile body site should be identified to the species level [14]. Additionally, CDC advises identifying *Candida* isolates recovered from non-sterile body sites to the species level when:

- Clinically indicated.
- *C. auris* has been detected in the facility or unit.
- A patient has had an overnight stay in a healthcare facility outside the United States in the preceding year, especially if that stay was in a country with documented *C. auris* transmission.

The presence of *C. auris* in any body site can represent a source for transmission and should trigger the implementation of infection control precautions [5].

Patients with *C. auris* colonization may also be identified through targeted screening. Screening may be considered when a patient is a close healthcare contact of someone with *C. auris* infection or colonization, or when a patient has had an overnight healthcare facility stay in a country outside the United States in the preceding year, especially if that country has documented *C. auris* cases [44]. Screening of patients with overseas healthcare exposure for *C. auris* is strongly encouraged when the patient has an infection or colonization with carbapenemase-producing Gram-negative bacteria. Point prevalence screenings, in which every patient on a unit or floor is screened at the same time, may be employed to detect unidentified colonized patients if there is evidence or suspicion of transmission in the facility [44]. Screening for *C. auris* is commonly done using a composite swab of the patient’s axilla and groin regions, as these sites have been determined to be high-yielding [40]. Other body sites or specimens from which *C. auris* has been isolated include the nose, mouth, external ear canals, urine, wounds, and rectum. The axilla and groin appear to be consistent sites of colonization [2,3,41], although further evaluation is needed. 

Hand hygiene: Healthcare personnel (HCP) should practice proper and frequent hand hygiene with alcohol-based hand sanitizer (ABHS) or soap and water. ABHS is effective against *C. auris* and is preferred for hand hygiene unless the hands are visibly soiled, in which cases handwashing with soap and water is recommended [40,45,46,47]. 

Care should be taken to ensure that enough quantities of ABHS, soap, towels, and uncluttered sinks are available in order to facilitate hand hygiene. HCP should be trained on appropriate hand hygiene techniques when hired and retrained at regular intervals. It is important to monitor HCP adherence with recommended hand hygiene practices and provide personnel with feedback regarding their performance.

Transmission-based precautions: All patients in acute care hospitals and long-term care hospitals who are infected or colonized with *C. auris* should be placed on contact precautions, which includes placing the patient in a single room and using appropriate personal protective equipment (PPE) and restricting patients to their room except for medically necessary procedures [40,47,48]. Whenever possible, patients with *C. auris* infection or colonization should be housed in a single-patient room. If a limited number of single rooms are available, they should be reserved for patients at highest risk for transmission, such as those with uncontained secretions or diarrhea. Patients colonized or infected with *C. auris* could also be cohorted in a room with other *C. auris* patients [49,50,51,52]. Cohorting can be challenging as *C. auris* patients are often co-colonized with other different multidrug-resistant organisms. This has made cohorting impractical in many settings (Table 2) [40].

Nursing homes may consider using less restrictive precautions if the patient’s unit is not experiencing ongoing transmission and if the resident does not have uncontained secretions or excretions. Enhanced barrier precautions have been recommended in these situations by CDC. As part of enhanced barrier precautions, PPE is used when body fluid exposure is anticipated as well as for high contact activities, such as dressing, device care, and changing linens, but is not required for other resident care activities. Under enhanced barrier precautions, residents are not restricted to their rooms and can participate in group activities [48].

In all settings, transmission-based precautions should be continued for as long as a patient is colonized with *C. auris.* The typical duration of *C. auris* colonization remains unknown, although it appears to be protracted while patients are in healthcare settings, and methods for decolonization are not yet established. Therefore, the most conservative strategy is to keep patients with *C. auris* infection or colonization on transmission-based precautions for the duration of their healthcare facility stays (present and future) [40]. To indicate that the patient is on transmission-based precautions and explain what PPE is needed, clear signage should be placed outside the patient’s room [40,48,49,52].

Environmental cleaning and disinfection: Extensive contamination of the healthcare environment has been described in facilities with *C. auris* outbreaks, emphasizing the importance of environmental cleaning and disinfection [45,53,54,55,56]. Environmental services staff should safely remove and clean visible organic material (e.g., bodily fluids, dirt) from patient care area before disinfection. Although quaternary ammonium compounds (QACs) are among the most commonly used disinfectants in healthcare settings, early studies found some of these compounds are ineffective against *C. auris* [45,53,54,55,56]. However, interpreting these studies is complicated because diverse methodologies have been utilized. In response, the Environmental Protection Agency (EPA) took two measures to improve disinfectant guidance for *C. auris*. First, CDC and EPA collaboratively implemented interim guidance for *C. auris* disinfection using EPA-registered hospital-grade disinfectants known to be effective against *Clostridioides difficile* spores until further data were available on efficacy of disinfectants against *C. auris*. Simultaneously, the EPA developed and released SOP-MB-35-00, a standardized quantitative disk carrier method that can be used to evaluate disinfectant efficacy against *C. auris*. 

Disinfectants that meet the 5 log_10_ reduction performance standard defined by the EPA can acquire a formal *C. auris* master label kill claim [45,53,54,55]. Recently the EPA has approved the addition of a *C. auris* claim to the master label of Oxivir 1 (applies to ready to use cleaners and wipes, EPA registration 70627-74 and 70627-77, respectively), a hydrogen peroxide based cleaner; and the Micro-Kill bleach germicidal bleach wipes (EPA registration 37549-1), a product based on sodium hypochlorite [54,57,58]. Additionally, the EPA has also approved a request made by CDC regarding a Section 18 emergency exemption (under the Federal Insecticide, Fungicide, and Rodenticide Act), which temporarily permits off-label use of seven additional disinfectants to control *C. auris*. This action is supported by efficacy data generated at CDC (personal communication D.J. Sexton, CDC) and expands the options available for healthcare facilities working to control *C. auris*. These developments represent helpful steps in expanding the number of disinfectants available for control of *C. auris*; however, further disinfectant testing and submissions for formal *C. auris* claims from the EPA are still needed. 

Work at CDC and in related publications by other groups have reaffirmed early concerns that some QAC-based products are not effective, but also indicated the promise of additional QAC chemistries that include alcohol-based products [59,60]. Thorough daily cleaning and disinfection, with special attention to high-touch surfaces such as bedrails and bedside tables, are needed in patient care areas housing patients on contact precautions for *C. auris*. Terminal cleaning and disinfection should be performed when the patient is moved from the care area permanently [40,47,49,61]. Chemical fogging, vaporized hydrogen peroxide, ozone, chlorine dioxide, and ultraviolet light, ionization, and titanium dioxide/ultraviolet light, might allow thorough disinfection of difficult-to-reach places, though further evaluations of these methods against *C. auris* are needed [40,47,49,61,62].

It is important to closely monitor adherence to environmental cleaning protocols, including protocols for cleaning solution preparation, contact times, designation of staff members’ assigned areas and objects to clean, and daily and terminal cleaning techniques. Routine environmental testing for *C. auris* is not recommended. Cultures are costly and time-intensive, and previous investigations have shown that *C. auris* will generally be detected in the environment where *C. auris* cases have been found [40]. Some facilities use machines for detection of adenosine triphosphate (ATP) to audit cleaning (not pathogen-specific testing), and standard environmental cleaning audits, such as direct observation or the use of florescent markings, to determine whether surfaces have been cleaned.

## 5. Treatment and Management of Infections and Colonization

It is highly recommended that *C. auris* infections be managed in consultation with an infectious disease specialist. Echinocandin drugs are recommended as initial therapy for treatment of *C. auris* infections, as *C. auris* isolates are often susceptible to echinocandins but are frequently resistant to the other two main antifungal drug classes (azoles and polyenes) [63]. Antifungal treatment management of *C. auris* infection is similar to other *Candida* species infections. More details on patients’ treatment and management are summarized in the Infectious Diseases Society of America (IDSA), Clinical Practice Guideline for the Management of Candidiasis published in 2016 [64]. 

No conclusive evidence exists regarding the effectiveness of protocols for the decolonization of patients with *C. auris* [40]. During an outbreak of *C. auris* in the United Kingdom, bathing with single-use wipes of 2% chlorhexidine gluconate (twice daily), or aqueous 4% chlorhexidine formulation were used on cases [65]. Patients on ventilator support also received mouth washing of 0.2% chlorhexidine or chlorhexidine 1% dental gel, and oral nystatin when oral oropharyngeal colonization was present. Additionally, chlorhexidine impregnated protective disks were used for all central vascular catheter exit sites to reduce line-associated *C. auris* BSIs [65]. Despite these efforts, *C. auris* colonization and transmission continued in the facility.

## 6. Communication

Communication of a person’s *C. auris* status is key to ensuring that infection control measures are carried out without disruption. When a patient is found to be infected or colonized with *C. auris*, appropriate communication and education are provided to HCP, so they understand the infection control protocols necessary. HCP should be made aware of the infection control requirements necessary for caring for a patient colonized or infected with *C. auris* and given sufficient resources to facilitate adherence. Information on *C. auris* infection or colonization should be communicated whenever patients are transferred to higher or lower levels of care so that the receiving facility is able to continue all infection control measures.

## 7. Conclusions

*C. auris* is an emerging multidrug-resistant pathogen that represents a serious threat to healthcare settings globally. This emerging pathogen presents unique issues related to rapid transmission, detection capacity, and specific environmental disinfection needs. However, many of the infection control procedures for *C. auris* represent standard and fundamental practices, such as hand hygiene or transmission-based precautions. Diligence in detection and infection control can help facilities prevent and control outbreaks of *C. auris.*

## Figures and Tables

**Table 1:** Common misidentifications of *Candida auris* when using on phenotypic identification, from CDC’s recommendations for identification of *Candida auris.*

Identification Method	Organism *C. auris* Can be Misidentified as
VITEK 2 YST *	Candida haemulonii Candida duobushaemulonii Software upgrade (version 8.01) includes *C. auris*. However, it is recommended to confirm isolates identified as *C. haemulonii* and *C. duobushaemulonii*, *C. famata* and *C. auris* by MALDI-TOF or DNA sequencing
API 20C	*Rhodotorula glutinis* (characteristic red color not present) *Candida sake*
BD Phoenix yeast identification system	*Candida haemulonii* *Candida catenulata*
MicroScan	*Candida famata* *Candida guilliermondii* *Candida lusitaniae* *Candida parapsilosis*
RapID Yeast Plus	*Candida parapsilosis*
Check databases of identification methods used, as capacity to detect *C. auris* may differ by database. * There have been reports of *C. auris* being misidentified as *Candida lusitaniae* and *Candida famata* on VITEK 2. A confirmatory test such as cornmeal agar may be warranted for these species.	
*C.guilliermondii, C. lusitaniae*, and *C. parapsilosis* generally make pseudohyphae on cornmeal agar. The absence of hyphae or pseudohyphae on cornmeal agar should raise the suspicion for *C. auris*. *C. auris* is able to grow at 40–42 °C with high salt concentrations (NaCl 10%). *C. auris* colonies appear white, pink, or red, and some colonies cannot be distinguished from *C. glabrata*. *C. auris* cannot be identified through morphology alone due to similarities with other *Candida* species. Supplemented or modified media has been shown to be useful for *C. auris* screening [7,8,9]. MALD-TOF and sequencing of D1-D2 region of the 28s rDNA or the internal transcribed region (ITS) of rDNA are recommended for an accurate identification of *C. auris* [12,13,14]. Table adapted from: https://www.cdc.gov/fungal/candida-auris/recommendations.html	

**Table 2:** Recommendations for infection control practices for *Candida auris*

**Identification of cases:** Identify the species of *Candida* isolated from sterile sites. Identify the species of *Candida* isolated from non-sterile sites when clinically indicated, when the patient resides on the facility or unit where a *C. auris* case has been identified, or when the patient had an overnight stay in a facility outside the United States in the past year, especially if in a country with *C. auris* transmission. Consider screening patients who: Are close healthcare contacts to new cases. Have had an overnight healthcare stay abroad in the past year, especially in a country with *C. auris* cases. This should be strongly considered when the patient is also infected or colonized with a carbapenemase-producing Gram negative bacteria [44]. If transmission is suspected, the healthcare facility should consider expanding screening to all individuals on the ward where cases have been identified. Infection control interventions are the same for patients with *C. auris* infection or colonization.
**Hand hygiene** Healthcare personnel (HCP) should practice proper and frequent hand hygiene. Monitor HCP adherence to hand hygiene practices and provide feedback.
**Transmission-based precautions** Place all patients infected or colonized with *C. auris* in acute care hospitals or long-term acute care hospitals on contact precautions. In nursing homes, consider placing residents with *C. auris* on less restrictive precautions (i.e., CDC’s enhanced barrier precautions), unless they have uncontrolled secretions or excretions or there is ongoing transmission on the unit or facility. Otherwise, use contact precautions. Patients appear to be persistently colonized long-term. Use of transmission-based precautions in healthcare settings should remain in place indefinitely. HCP adherence to transmission-based precautions should be frequently monitored. Use signage to indicate patient are on transmission-based precautions. Signage should be placed in a visible area and clearly indicate what precautions and PPE are required.
**Environmental cleaning** Use registered hospital-grade disinfectant effective against *Clostridioides difficile* spores [54,55]. Three products have recently acquired efficacy claims against *C. auris*: Environmental Protection Agency (EPA) registration: 70627-74, 70627-77 and 37549-1 [57,58]. Disinfectants based solely on quaternary ammonium compounds are generally ineffective against *C. auris* [45,53]. Thorough daily and terminal cleaning and disinfection are needed in *C. auris* patient care areas. Shared medical equipment should be cleaned and disinfected thoroughly. Monitor environmental cleaning and disinfection adherence.
**Patient decolonization** There is currently no established protocol for the decolonization of patients with *C. auris.*
